# Patient-Derived Ovarian Cancer Spheroids Rely on PI3K-AKT Signaling Addiction for Cancer Stemness and Chemoresistance

**DOI:** 10.3390/cancers14040958

**Published:** 2022-02-15

**Authors:** Deepak Parashar, Anjali Geethadevi, Sonam Mittal, Lindsey A. McAlarnen, Jasmine George, Ishaque P. Kadamberi, Prachi Gupta, Denise S. Uyar, Elizabeth E. Hopp, Holli Drendel, Erin A. Bishop, William H. Bradley, Kathleen M. Bone, Janet S. Rader, Sunila Pradeep, Pradeep Chaluvally-Raghavan

**Affiliations:** 1 Department of Obstetrics and Gynecology, Medical College of Wisconsin, Milwaukee, WI 53226, USA; dparashar@mcw.edu (D.P.); ageethadevi@mcw.edu (A.G.); smittal@mcw.edu (S.M.); lmcalarnen@mcw.edu (L.A.M.); jasgeorge@mcw.edu (J.G.); ipulikkal@mcw.edu (I.P.K.); prgupta@mcw.edu (P.G.); duyar@mcw.edu (D.S.U.); ehopp@mcw.edu (E.E.H.); erbishop@mcw.edu (E.A.B.); wbradley@mcw.edu (W.H.B.); jrader@mcw.edu (J.S.R.); spradeep@mcw.edu (S.P.); 2 Department of Pathology, Medical College of Wisconsin, Milwaukee, WI 53226, USA; hdrendel@mcw.edu (H.D.); kbone@mcw.edu (K.M.B.); 3 Department of Physiology, Medical College of Wisconsin, Milwaukee, WI 53226, USA; 4 Cancer Center, Medical College of Wisconsin, Milwaukee, WI 53226, USA

**Keywords:** epithelial ovarian cancer, cancer stemness, spheroids, PI3K/AKT signaling, signaling addiction, cancer cell line development

## Abstract

**Simple Summary:**

Epithelial ovarian cancer (EOC) is the most fatal gynecological cancer with poor survival rates and high mortality. EOC patients respond to standard platinum-based chemotherapy in the beginning, but relapse often due to chemoresistance. Ovarian cancer cells disseminate from the ovarian tumors and spread within the abdomen, where ascites fluid supports the growth and transition. Malignant ascites is present in a third of patients at diagnosis and is considered as a major source of chemoresistance, recurrence, poor survival, and mortality. Malignant ascites is a complex fluid that contains a pro-tumorigenic environment with disseminated cancer cells in 3D spheroids form. In this study, we established an ovarian cancer cell line and identified that 3D spheroids develop from the 2D monolayer, and the platinum-resistant phenotype develops due to the aberrant PI3K-AKT signaling in tumor cells. Furthermore, when we used a combinatorial approach of cisplatin with LY-294002 (a PI3K-AKT dual kinase inhibitor) to treat the cisplatin version of both MCW-OV-SL-3 and A-2780 cell lines, it prevented the 3D spheroid formation ability and also sensitized the cells for cisplatin. In brief, our results provided evidence to advance therapeutic approaches to treat cisplatin resistance in ovarian cancer patients.

**Abstract:**

Ovarian cancer is the most lethal gynecological malignancy among women worldwide and is characterized by aggressiveness, cancer stemness, and frequent relapse due to resistance to platinum-based therapy. Ovarian cancer cells metastasize through ascites fluid as 3D spheroids which are more resistant to apoptosis and chemotherapeutic agents. However, the precise mechanism as an oncogenic addiction that makes 3D spheroids resistant to apoptosis and chemotherapeutic agents is not understood. To study the signaling addiction mechanism that occurs during cancer progression in patients, we developed an endometrioid subtype ovarian cancer cell line named ‘MCW-OV-SL-3’ from the ovary of a 70-year-old patient with stage 1A endometrioid adenocarcinoma of the ovary. We found that the cell line MCW-OV-SL-3 exhibits interstitial duplication of 1q (q21–q42), where this duplication resulted in high expression of the PIK3C2B gene and aberrant activation of PI3K-AKT-ERK signaling. Using short tandem repeat (STR) analysis, we demonstrated that the cell line exhibits a unique genetic identity compared to existing ovarian cancer cell lines. Notably, the MCW-OV-SL-3 cell line was able to form 3D spheroids spontaneously, which is an inherent property of tumor cells when plated on cell culture dishes. Importantly, the tumor spheroids derived from the MCW-OV-SL-3 cell line expressed high levels of c-Kit, PROM1, ZEB1, SNAI, VIM, and Twist1 compared to 2D monolayer cells. We also observed that the hyperactivation of ERK and PI3K/AKT signaling in these cancer cells resulted in resistance to cisplatin. In summary, the MCW-OV-SL3 endometrioid cell line is an excellent model to study the mechanism of cancer stemness and chemoresistance in endometrioid ovarian cancer.

## 1. Introduction

Ovarian cancer is the fifth leading cause of cancer-related mortality and the 11th most common cancer in women. In developing countries, the mortality rate in ovarian cancer patients is even higher due to challenges in early detection, poor prognosis, frequent relapses, lack of medical infrastructure, and limited awareness [1]. As compared to other malignancies, early-stage ovarian cancer is often asymptomatic, and therefore spreads throughout the abdominal cavity with most patients presenting later with an advanced stage ovarian cancer. Histologically, epithelial ovarian cancer is a heterogenous disease of five pathologically distinct subtypes such as (1) high-grade serous ovarian carcinoma (HGSOC), (2) low-grade serous (LGS), (3) endometrioid, (4) clear cell, and (5) mucinous carcinoma subtypes. Among these subtypes, HGSOC is the most prevalent subtype representing approximately 75% of cases, while the remaining 25% are made up of low-grade serous (LGS), endometrioid, clear cell, and mucinous carcinoma [2]. Therefore, the majority of research is focused on cell lines particularly of serous ovarian carcinoma origin, and the other histologic subtypes of ovarian cancer are poorly represented in the available list of ovarian cancer cell lines [2]. The most commonly used cell lines for serous ovarian cancer are SKOV-3, OVCAR-3, OVCAR-4, CAOV3, HEYA8, and IGROV1. Unfortunately, some of these cancer cell lines, which have been used for many years, are misidentified, or misclassified histologically [2]. Another concern pertaining to the use of these cell lines in ovarian cancer research arose due to the misrepresentation of the status of p53 and BRCA1 as wild type or in its mutated form [3].

In contrast to other cancers, peritoneal seeding is considered as the primary mechanism of metastatic spreading, while metastasis through the hematogenous mode is much more limited [4]. Accumulation of ascites is also a common feature in ovarian cancer, which consequently worsens the prognostic outcome of ovarian cancer. Notably, various growth factors, cytokines, chemokines, extracellular matrix proteins, and proteolytic enzymes promote the growth and progression of ovarian cancer by activating varieties of oncogenic pathways in tumor cells [5]. Ovarian cancer metastasis relies on the process where tumor cells from ovary shed into the peritoneum, then aggregate as freely floating spheroids which get implanted on various peritoneal organs [6,7]. As compared to monolayers, tumor spheroids derived from monolayer cells demonstrate resistance to cytotoxic drugs often due to hinderance of drug penetration, induction of cellular efflux pumps, and aberrant activation of oncogenic signaling pathways in tumor spheroids [8,9,10]. Therefore, ovarian cancer cell lines, which form tumor spheroids spontaneously when confluent will have enormous value for studying the mechanism of transition from adherent form to tumor spheroids, drug resistance, anoikis, and metabolic changes. Thus, there is an urgent need to identify signaling mechanisms distinct in tumor spheroids, which are more aggressive in nature for identifying suitable targeted therapy to be used alone or in combination with chemotherapeutic agents.

Cancer cells with aggressive characteristics such as high-proliferative capacity, metastatic features, or chemoresistance rely on a precise signaling mechanism through autocrine or paracrine cues as an oncogenic signaling addiction. Therefore, we sought to characterize the precise signaling that promotes cancer stemness and the chemoresistance mechanism in one of the common subtypes of ovarian cancer endometrioid histologic subtype. Endometrioid ovarian carcinoma constitutes about 20% of epithelial ovarian carcinoma among women in the United States and commonly arises from endometriosis of the ovary [11]. Patients with endometrioid carcinoma of the ovary are often diagnosed at a comparatively younger age with early-stage disease and have relatively better 5-year survival outcome (80.3%) than serous ovarian carcinoma (18.4%). By establishing an endometrioid subtype cell line, we uncovered the mechanism required for spontaneous transition of monolayer cells to tumor spheroids and chemoresistance in endometrioid subtype cancer cells.

## 2. Material and Methods

### 2.1. Patients and Human Ethics Statement

Written informed consent was obtained from the patient according to the Institutional Review Board (IRB) of the Medical College of Wisconsin, Milwaukee, WI, USA. A 70-year-old female presented with elevated CA-125 of 81.8 units/mL, and a complex right adnexal mass. She underwent staging surgery including exploratory laparotomy, total abdominal hysterectomy, bilateral salpingo-oophorectomy, omentectomy, diaphragm cytology, appendectomy, pelvic and para-aortic lymph node dissection. The patient was untreated at the time of surgery. On the histopathology examination, the right ovary was positive for adenocarcinoma, endometrioid type (FIGO stage 1A, grade 1) with extensive necrosis. The left ovary and fallopian tube were negative for carcinoma. All lymph nodes (left and right pelvic and para-aortic lymph nodes) were negative for carcinoma.

### 2.2. Isolation and Culture of Ovarian Tumor-Derived Cells

**Figure 1:**
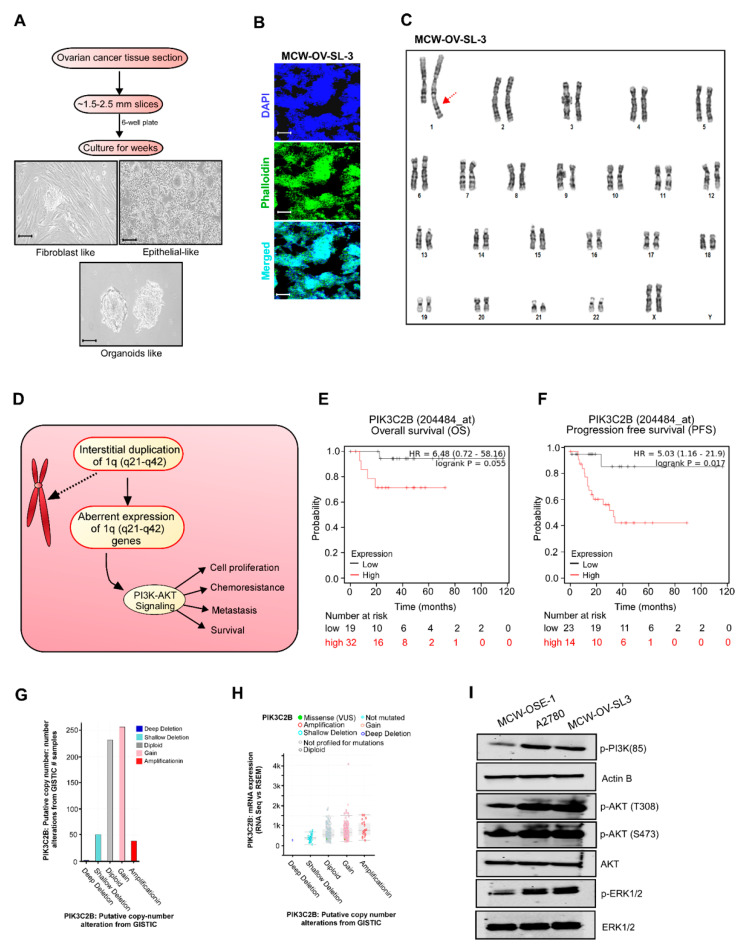
Establishment and characterization of the MCW-OV-SL3 cell line. (**A**) Workflow demonstrates the establishment of MCW-OV-SL-3 cell lines from endometroid ovarian cancer tissue. Isolated tissue sections were cut into small ~1.5–2.5 mm slices, then cultured in DMEM medium for 4 weeks until small organoids were formed. Fibroblasts were eliminated by differential trypsinization method until immortalized MCW-OV-SL-3 were grown continuously. Scale bars represent 500 µm (**B**) MCW-OV-SL3 cells were immune stained using nucleus stain DAPI (Blue) and Phalloidin (green) for F-actin and photographed using a fluorescent microscope. Scale bars represent 500 µm (**C**) Representative karyogram of MCW-OV-SL3 cell lines. Red arrow indicates duplication in the 1q locus (q21–q41). (**D**) A schema proposes how PIK3C2B duplication at the 1q locus (q21–q41) causes aberrant PI3K-AKT signaling. (**E**,**F**) Overall survival and progression free survival analysis in endometroid ovarian cancer. Patient samples that were stratified as high versus low based on median expression of PIK3C2B expression using KM survival analysis plotter. (**G**) Copy number variation (CNV) analysis was performed based on the copy number of PIK3C2B in ovarian cancer patients using GISTIC software from TCGA datasets. (**H**) mRNA expression analysis of PIK3C2B was performed based on the RNA seq data in ovarian cancer patients using GISTIC software from TCGA datasets. (**I**) Cell lysates were prepared from MCW-OSE-1, A2780, and MCW-OV-SL-3 monolayer cells and immunoblot was performed with indicated antibodies (see Figure S3).

Tumor tissue obtained was minced into approximately 1.5–2.5 mm^3^ pieces, washed with 1X PBS (Ca^2+^ and Mg^2+^ free), then digested using 0.1% Collagenase Type IV (Gibco, ThermoFisher Scientific, Waltham, MA, USA) in DMEM for 30 min at 37 °C with occasional shaking at low speed in a 15 mL centrifuge tube (Corning Inc., Corning, NY, USA). The cell suspension was filtered by 45 µm cell strainers to remove any large tissue fragments and centrifuged at 1000 rpm for 3 min. Cells were resuspended in advanced DMEM supplemented with 10% fetal bovine serum (FBS, R&D Systems, Inc., Minneapolis, MN, USA), 50 units/mL penicillin, and 50 μg/mL streptomycin (Gibco, ThermoFisher Scientific, Waltham, MA, USA), 2 mM L-glutamine (Gibco, Thermo Fisher Scientific, Waltham, MA, USA), and cultured in 24-well cell culture plates at 37 °C in a humidified 5% CO_2_ atmosphere. Continuous culture resulted in adhesion to the culture dishes and an outgrowth of cells attached to the tissue and the plate was observed daily (Figure 1). Two types of cells, epithelial-like cuboidal cells and fibroblast-like elongated cells were visible for the few weeks. Fibroblast cells were eliminated by differential trypsinization method. Later, epithelial-like cells started making dome-like colonies. These cell domes were isolated, picked, and transferred to a fresh tissue culture flask. These cells were continuously passaged 40 times for about 6 months, and a tumor cell clone was manually selected to establish the stable MCW-OV-SL-3 cell line. The cells were frozen in 5% DMSO in fetal bovine serum (FBS) solution in liquid nitrogen for further experiment. Karyotyping was performed after the 15th passage for characterization. These cells were regularly passaged and expanded in complete DMEM media for characterization. During early steps of establishment, this cell line grew rapidly in the presence of 2% heat inactivated autologous human ascitic filtrate. Once the cell line was established the filtrate was no longer added. Short tandem repeat (STR) characterization was performed at IDEXX Bioanalytics Services (Columbia, MO, USA) for cell authentication.

### 2.3. Isolation and Culture of Normal Ovarian Surface Epithelial (OSE) Cells

Normal ovarian surface epithelium cells (OSE) were obtained by scraping the surface of healthy ovaries from non-cancerous patient from the Department of Obstetrics and Gynecology, Froedtert Hospital, Medical College of Wisconsin. All human samples were collected with written informed consents from patients under an IRB of the Medical College of Wisconsin-approved protocol in accordance with recognized ethical guidelines of the declaration of Helsinki.

Histological examination confirmed that ovaries were grossly normal, and no pathological lesions were observed. The normal ovarian tissue obtained was minced into approximately 1.0–2.0 mm^3^ pieces, then washed with 1X PBS. The OSE cells were scraped with a surgical blade after collagenase digestion under aseptic conditions using 0.1% Collagenase Type IV (Gibco) in DMEM for 30 min at 37 °C with occasional shaking at low speed in a 15 mL centrifuge tube (Corning Inc., Corning, NY, USA). Then, cell viability was checked by the trypan blue dye exclusion assay, and it was observed that 95% cells were viable. OSE cells isolated were cultured with Medium 199/MCDB105 (1:1, Sigma, St. Louis, MO, USA). supplemented with 15% FBS, 1% pen-strep, 10 ng/mL human epidermal growth factor (Life Technologies, Carlsbad, MA, USA), 0.5 μg/mL hydrocortisone (Sigma St. Louis, MO, USA)., 5 μg/mL bovine insulin (Cell Applications, CA, USA), 34 μg protein/mL bovine pituitary extract (Life Technologies, Carlsbad, MA, USA) at 37 °C in a humidified 5% CO_2_ atmosphere. Cells were detached from the dish with 0.125% trypsin and 0.11% ethylenediamine tetra acetic acid (EDTA) and split in 1:2 ratios in a new culture dish when confluent.

Karyotyping was performed after the 5th passage for characterization and STR profiling was performed at IDEXX Bioanalytics Services (Columbia, MO, USA) to confirm that the identity of the cell line we established was unique and was not contaminated with other established cell lines.

### 2.4. Cell Lines

A2780 parental and cisplatin-resistant A2780-cisR cells were obtained from Millipore Sigma (St. Louis, MO, USA). The OVCAR-4 cell line was purchased from NCI-DCTD repository. The HeyA8 cell line was received from the Characterized Cell Line core at M.D. Anderson Cancer center, Texas, USA. All cell lines were cultured in Dulbecco’s DMEM high glucose (4.5 g/L) supplemented with 10% fetal bovine serum, 2 mM L-glutamine, and antibiotics (60 mg/L penicillin, 100 mg/L streptomycin sulfate (PenStrep)) at 37 °C under 5% CO_2_. A2780-cisR cells were maintained in the presence of 2 μM of cisplatin. Cell line authentication was performed by short tandem repeat profiling at the IDEXX Bioanalytic Laboratories Inc. and tested as Mycoplasma negative by PCR detection kit (Mycosenser Mycoplasma assay kit, Agilent, Santa Clara, CA, USA) as recent as two months prior to the previous experiments.

Cisplatin-resistant (CisR) variants of the MCW-OV-SL-3 cell line were derived in vitro from their original parental (PT) cell line by continuous exposure to cisplatin (Selleckchem, Houston, TX, USA) before intraperitoneal injection. Initially, the MCW-OV-SL-3-CisR subline was treated with cisplatin (IC50 of 2.5 uM) for 72 h. To recover the cells, the old media with cisplatin was changed with complete DMEM without cisplatin and cells were left for a further 72 h. This development period continued with increasing doses of cisplatin for approximately 12 months, after which the IC50 concentrations were re-assessed in the resistant cell line. The resistant cells were maintained with new IC-50.

### 2.5. Karyotype Analysis

Karyotyping was performed on MCW-OSE-1 (ovarian surface epithelial cells from normal ovary of healthy control) and MCW-OV-SL-3 cell lines to determine the chromosomal abnormalities as described before [12]. Cells in the logarithmic growth phase were cultured in DMEM until 80% confluency and were sent to Wisconsin Diagnostic Laboratories Cytogenetics for karyotyping.

Metaphase spreads were prepared following standard cytogenetic procedures. Briefly, cells were harvested by trypsinization and complete DMEM was added to inactivate trypsin. After centrifugation at 1000 rpm for 4 min, cells were initially exposed to 50 µL of EtBr (0.33 mg/mL) for 1 h, followed by 100 µL of 1.11 µg/mL colcemid for 20 min and fixed in Carnoy’s fixative (1:3 acetic acid: methanol) twice at room temperature for 30 min. Slides were air-dried and stained with Giemsa stain for 20 min. Twenty metaphase cells from each cell line were analyzed and 3–4 metaphase spreads were karyotyped according to the International System for Human Cytogenetic Nomenclature (ISCN 2020) [13,14]. The karyotyping was performed after analyzing 20 proliferating (metaphases) cells with 400–550 band resolution in MCW-OSE-1 and 400–425 band resolution in MCW-OV-SL-3 cells.

### 2.6. Drugs

Cisplatin (NSC 119875, Cisplatinum, cis-diamminedichloroplatinum II, CDDP, cis DDP, DDP) was purchased from Selleckchem and a 5 mM stock solution was prepared. LY294002 (S1105, NSC 697286) was purchased from Selleckchem. Aliquots of cisplatin and LY294002 were stored at −20 °C for up to a maximum of three months and thawed immediately before use.

### 2.7. Morphological Examination of MCW-OSE-1 and MCW-OV-SL-3

MCW-OSE-1 and MCW-OV-SL-3 cells were seeded in a 6-well tissue culture plate and incubated at 37 °C in a humidified 5% CO_2_ incubator for 2 weeks. Cells were observed under phase contrast microscope daily to check general morphology.

### 2.8. 3D Spheroid Formation Assay

Spheroid formation (3D culture) with 3000 cells per spheroid was performed as described previously [15]. In brief, a cell monolayer was washed with 1X PBS and treated with trypsin–EDTA solution for 2 min at 37 °C. Trypsin was then neutralized by adding complete growth medium, and the cell suspension was centrifuged at 1000 rpm for 4 min. Supernatant was removed and 3000 cells per spheroid were resuspended in 500 μL of complete DMEM containing 5% growth factor reduced Matrigel (BD Biosciences, Bedford, MA, USA) in each well of 24-well, growth factor reduced Matrigel-coated non-adherent plates for 7 days. In parallel, cells were also seeded on cell culture plates (2D culture) and cultivated in the same medium and incubated at 37 °C in a 5% CO_2_ incubator. Cultures were treated with different inhibitors (either cisplatin (2.2 and 6.2 µM), LY-294002 (5 and 10 µM) or their combination (either cisplatin 1.1 and 3.1 µM), LY-294002 (2.5 and 5 µM) for 48 h. Morphology of the monolayer and spheroids was evaluated using a phase contrast microscope and number of spheroids was determined.

### 2.9. Cell Viability Assay

To measure the cell viability, MCW-OSE-1, MCW-OV-SL3, OVCAR 8, OVCAR 4, SKOV3, and A2780 cells were seeded at the density of 5 × 10^3^ cells/well with five replicates in 96-well plates. The next day, after reaching 80% confluency, the cells were treated with cisplatin, PI3K inhibitor (LY294002), or a combination of cisplatin and LY294002. Cell viability was measured using 3-(4,5-dimethylthia-zol-2-yl)-2,5-diphenyltetrazolium bromide (MTT) reagent (Sigma Aldrich, St. Louis, MO, USA). MTT reagent (5 mg/mL) was added into each well of 96-well plates and cells were incubated at 37 °C for 3 h. Formazan crystals thus formed were dissolved with acidic isopropanol, and absorbance was measured at 560 nm in a microplate reader (Tecan, Mannedorf, Switzerland). Cell viability at 24, 48, and 72 h was calculated by taking the ratio to control cells from the day 0 reading to account for plating unevenness.

### 2.10. Cellular Proliferation and Colony Formation Assay

To study the cellular proliferation, 1 × 10^5^ cells were seeded in tetraplicates in a 6-well plate. Cell number was counted manually with a hemocytometer at three different time points after seeding for 24, 48, and 72 h. For the colony formation assay, 1000 cells per well were seeded in a 6-well plate in triplicates. Cell were also treated with cisplatin, LY294002, or a combination of cisplatin and LY294002 for 48 h before seeding and incubated at 37 °C in a 5% CO_2_ incubator. After 14 days, cells were rinsed with 1X PBS, fixed in 5% glutaraldehyde for 20 min, and stained with 0.5% crystal violet (Sigma Aldrich, St. Louis, MO, USA) for 20 min. Plates were washed with water and dried before scanning. Crystal Violet was solubilized with 10% acetic acid and absorbance was measured at 450 nm in a microplate reader (Tecan, Mannedorf, Switzerland).

### 2.11. Cell Migration, Invasion, and Wound Healing Assay

The cellular motility was analyzed by carrying out cell migration, invasion, and wound healing assay as described earlier [10]. Cells were also treated with cisplatin, LY294002, or a combination of both for 48 h before migration, invasion, and wound healing assays. In brief, for the migration assay, 1 × 10^5^ cells were seeded on the upper chamber of the 8.0 µm pore trans-well inserts (BD Biosciences, Bedford, MA, USA). Growth medium containing 10% FBS was the chemoattractant in the lower chamber.

For the invasion assay inserts were coated with 5 mg/mL Matrigel (BD Biosciences, Bedford, MA, USA) and 2 × 10^5^ cells were seeded similarly as for the migration assay. Cells were incubated at 37 °C for 12 h for migration and invasion assays and fixed with 0.5% glutaraldehyde for 20 min. Non-migrated cells were removed using a cotton swab and culture inserts were washed and stained with 0.5% crystal violet in 10% methanol. Migrated and invaded cells were imaged using a phase contrast microscope. Moreover, stained membranes were also dissolved in 10% acetic acid and absorbance was measured in a microplate reader at 560 nm.

For the wound healing assay, 2 × 10^6^ cells were seeded in a 35 mm Petri dish. After 12 h of incubation, a wound was mechanically created using an aerosol P200 pipette tip, and cells were photomicrographed at various time points using a phase contrast micro-scope (Nikon, Fukok, Japan). Images were analyzed using Image J software (https://imagej.nih.gov/ij/download.html accessed on 20 August 2021).

### 2.12. RNA Isolation, cDNA Synthesis, and Real-Time PCR Analysis

Total RNA from cancer cells and normal ovarian cells were extracted using RNeasy Plus kit (QIAGEN, Valencia, CA, USA) according to the manufacturer’s protocol. mRNA level of various genes was determined in a Bio-Rad CFX Connect using SYBR Green Supermix (Bio-Rad, Hercules, CA, USA). Primers for real-time PCR were designed using primer3 software (https://bioinfo.ut.ee/primer3-0.4.0/ accessed on 30 April 2021) and listed (Supplementary Table S1). PCR was performed as follows: hot start for 2 min at 95 °C, denaturation for 10 s at 95 °C, annealing for 15 s according to the Tm of each primer, and extension for 10 s at 72 °C for 15–30 cycles. Relative mRNA level was quantitated using β-actin or GAPDH as an endogenous control using the ΔΔCt algorithm. The experiment was performed in technical and experimental triplicates. RT2 Profiler PCR Array for genes associated with tumor cell proliferation, EMT, and cancer stemness (catalog no., CAPA9696-12: CLAH36595) was purchased from Qiagen. 

### 2.13. Immunoblot Analysis

Immunoblotting was performed as described earlier with some modification [16,17,18]. Briefly, whole-cell lysates were prepared from 2D or 3D cultures. Cells and spheroids were washed with cold 1X PBS and lysed using RIPA lysis buffer (50 mM Tris-HCl pH 7.6, 150 mM NaCl, 1% TritonX-100, 0.1% SDS, 0.5% sodium deoxycholate, 1 mM Phenylmethylsulfonyl fluoride (PMSF), 4 μg/mL aprotinin, 4 μg/mL leupeptin, 0.6 μg/mL benzamidinchloride, 20 μg/mL trypsin inhibitor) for 15 min at 4 °C. Lysates were centrifuged at 4 °C for 15 min at 13,000 rpm to remove insoluble debris. Protein concentration in the lysate was estimated using bicinchoninic acid (BCA method) as described by the manufacturer (Thermo Fisher Scientific Inc., Waltham, MA, USA). Proteins were then resolved on a 10% SDS-PAGE gel and transferred to a polyvinylidene difluoride membrane (Millipore Corporation, Burlington, MA, USA). The PVDF membrane was blocked with 5% nonfat dry milk and was incubated with primary antibody (1:1000 dilution) overnight at 4 °C followed by incubation with horseradish peroxidase-conjugated secondary antibodies (Bio-Rad, Hercules, CA, USA). β-actin was used as a loading control. The expression of specific proteins was detected using chemiluminescence in iBright Western Blot Imaging Systems (Thermofisher scientific, Waltham, MA, USA). For antibodies, see Table S2.

### 2.14. Animal Study

All experiments on mice were performed in accordance with the Medical College of Wisconsin institutional Animal Care and Use Committee. All mice were housed and cared for according to the Institutional Animal Care and Use Committee (IACUC) at the Medical College of Wisconsin with institution guidelines. Animal health was monitored daily, and animal weights measured at least weekly.

#### Subcutaneous Tumor Cell Inoculation

MCW-OV-SL-3 or A2780 cells were trypsinized, washed, and resuspended in Hanks’ balanced salt solution (HBSS, GIBCO, Carlsbad, CA, USA) and a 50 µL cell suspension containing 1 × 10^6^ cells was injected into each mouse (*n* = 6) subcutaneously into the left flank region. We purchased 4–6-week-old female athymic nude mice (CrTac: NCr-*Foxn1^nu^*) from Taconic Laboratories and they were housed in pathogen free conditions. Tumor-bearing mice were randomly divided into four groups (*n* = 6/group) after tumors had grown to an average of 100 mm^3^. Mice were treated weekly with intraperitoneal doses of PBS control, cisplatin (7 mg/kg body weight) at room temperature beginning on day 10 post-inoculation. Tumor volume was calculated according to the formula V = (length × width 2)½. Treatment was continued for 7 weeks, at which point, all mice were sacrificed, necropsied, and tumors were harvested. Subcutaneous tumor measurement was performed weekly in mice exhibiting palpable subcutaneous tumors until 6 weeks or humane endpoints. Tumor tissue was prepared as snap frozen for RNA and protein isolation or fixed in 10% formalin for immunohistochemistry.

### 2.15. Bioinformatic Analysis

A heatmap representing fold change of genes in monolayer versus spheroids was prepared by the Qiagen RT-PCR Profiler Software (https://dataanalysis2.qiagen.com/pcr accessed on 5 March 2021) by converting the individual normalized ΔΔCt values of “monolayer” and “spheroid” population to 2^−ΔΔCt^. The experiment was performed in technical triplicates as recommended by the Qiagen RT-PCR Profiler Analysis program. Genes with ≥ 1.4-fold change in expression with a *p* < 0.05 were selected. Five housekeeping genes (B2M, HPRT1, RPLP0, GAPDH, and ACTB) were used for normalizing the data.

Clinical Data Analysis was performed by cBioPortal http://www.cbioportal.org/ accessed on 8 April 2021 and GISTIC2 (Genomic Identification of Significant Targets in Cancer, version 2) analysis using Firehose-suggested parameters. Protein–protein interaction was analyzed by String software (Search Tool for the Retrieval of Interacting Genes/Proteins, https://string-db.org/ accessed on 15 March 2021 ).

### 2.16. Statistical Analysis

All assays were performed in at least triplicate or more as indicated in the figure legends. Data are represented as means ±SE. Statistical comparisons were performed using unpaired two-tailed Student’s t tests or by ANOVA, where appropriate, with a probability value of 0.05 considered significant using GraphPad Software https://www.graphpad.com/scientificsoftware/prism/ accessed on 19 March 2020.

## 3. Results

### 3.1. Ovarian Cancer Cells Established from the Tissue of Ovarian Cancer Patient Demonstrated Spheroid Forming Ability

We successfully established an immortalized ovarian cancer cell line, MCW-OV-SL-3, and a primary normal ovarian cell line MCW-OSE-1. The MCW-OV-SL-3 cell line was derived from a 70-year-old patient diagnosed with stage 1A, grade 1 endometrioid ovarian cancer. Single cells were prepared from the ovarian tumor tissue and normal ovarian epithelium was passaged for the selection of epithelial cells. Ovarian cancer cells formed as spheroids which were selected and propagated further as single cells, which again formed as a spheroid by budding from the monolayer of epithelial cells (Figure 1A,B). Short tandem repeat (STR) analysis confirmed that MCW-OV-SL-3 cell line is human derived and shows a unique STR identity compared to any other established cell lines (Figure 1C and Figure S1A,B).

### 3.2. Genome Analysis of MCW-OV-SL-3 Cell Line Exhibited 1q Locus (1q21–q42) Duplication and Aberrant PIK3C2B Expression and PI3K-AKT Signaling

Next, we performed karyotyping analysis to evaluate the number and structure of chromosomes in the MCW-OV-SL3 cell line. Chromosomes of 20 proliferating cells were counted and fully analyzed using G-banding and three cells were karyotyped. These cells had a modal number of 46 chromosomes including a pair of female X chromosomes. Notably, we found that 20 analyzed cells of MCW-OV-SL-3 demonstrated an interstitial duplication in the 1q locus (q21–q42) (Figure 1C). In parallel we established MCW-OSE-1 from normal ovarian surface epithelial cells, to compare with the oncogenic potential of MCW-OV-SL3 cell lines. Our karyotypic analysis of MCW-OSE-1 cells did not show any abnormalities in the chromosomal number or banding patterns. We also noticed that these cells had a modal number of 46 chromosomes including a pair of female X chromosomes (Figure S1B).

It is well reported that PIK3C2B is located on the 1q32 locus and plays an important role in several process including cell survival, metabolism, motility, and is one of the most frequently deregulated pathways in human cancer (Figure 1D). Further we analyzed percentage alteration frequency of different genetic and transcriptional alterations of PIK3C2B pathway elements such as AKT1, AKT2, AKT3, PIK3C2B, PIP5K1A, PIP5K1B, PIP4K2A, PIP4K2B, and PI4KB, etc., in HGSOC tissues and other cancer tissues and PIK3C2B protein abundance using cBioPortal and TCGA datasets. Interestingly we found that AKT3 and PIK3C2B were 32% and 27% significantly higher, respectively, in ovarian cancer datasets (Figure S1C). Further, the ovarian cancer dataset from Kaplan Meier Plotter was used to test for the survival prediction capacity of PIK3C2B, and we found that PIK3C2B is associated with poor overall survival and progression free survival in endometrioid ovarian cancer patients but not in HGSOC (Figure 1E,F and Figure S1D). Next, we checked the putative copy number alteration and mRNA expression of PIK3C2B from GISTIC software and found a significant trend of PIK3C2B gain and amplification in ovarian cancer patients (Figure 1G,H). We also observed that PIK3C2B is highly amplified in ovarian cancer patients and ovarian cancer is one of the cancers where PIK3C2B was expressed in modest to high level in the TCGA dataset (Figure S1E,F). Further, the evidence of PIK3C2B log2 expression through DepMap software https://depmap.org/portal/ accessed on 8 April 2021 in different primary and metastatic ovarian cancer cell lines also supports that PIK3C2B is significantly greater in ovarian cancer cell lines (Figure S1G). The PIK3C2B gene belongs to the phosphoinositide 3-kinase (PI3K) family. PI3K-AKT signaling is also known for its oncogenic effects including increased cell proliferation, oncogenic transformation, metastasis, chemoresistance, and intracellular protein trafficking [19]. In conjunction, we observed that endometroid ovarian cancer cell line MCW-OV-SL-3 cells as well as A2780 exhibited increased phosphorylation of AKT both at T308 and S473 residues, phosphorylation of p85 subunit of PI3K, and phosphorylation of ERK1/2 compared to normal MCW-OSE-1 cells (Figure 1I). Notably, we found that the level of phosphorylation of AKT both at T308 and S473 residues and phosphorylation of ERK1/2 in MCW-OV-SL3 cells were equivalent to the levels in A2780 ovarian cancer cells (Figure 1I).

### 3.3. MCW-OV-SL-3 Cell Line Exhibited the Characteristics of Aggressively Growing Ovarian Cancer Cells

**Figure 2:**
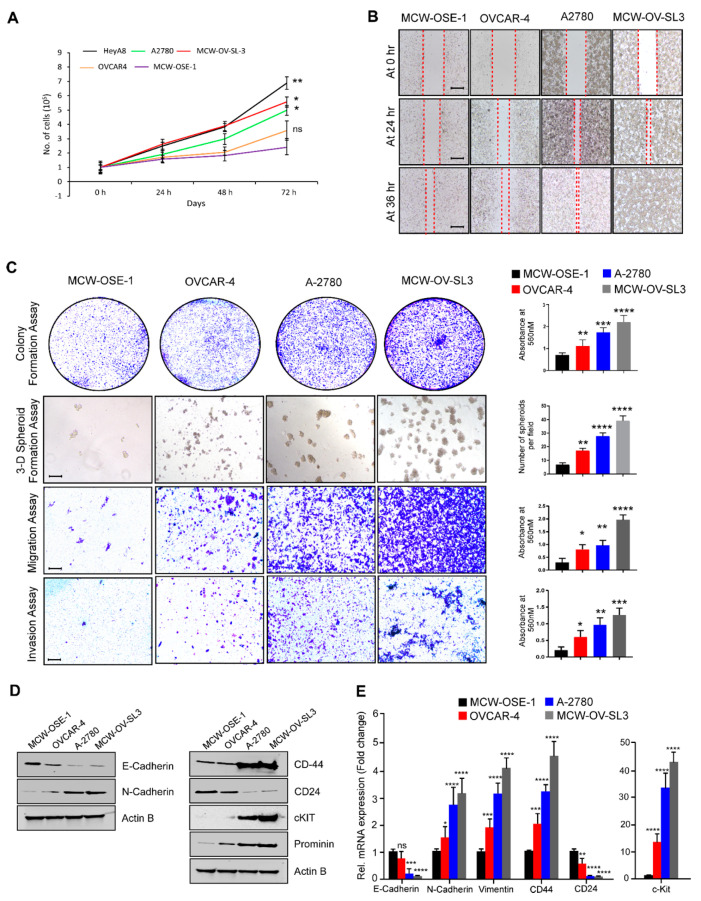
MCW-OV-SL3 cell line exhibits oncogenic characteristics similar to the aggressive type of ovarian cancer cells. (**A**) MCW-OSE-1, MCW-OV-SL3, and the cell lines indicated were grown on 96-well culture plates for indicated periods and the cell proliferation was assessed using manual counting. (**B**) Cell lines were grown on 6-well culture plates for 14 days for colony formation, then stained using 0.5% crystal violet and photographed. For 3D formation assay, cells were grown on Matrigel-coated plates for seven days and then photographed using phase-contrast microscope. Spheroids were counted from five separate fields and quantitated (right panel). For cell migration and invasion, cells were plated on trans-well inserts with or without Matrigel for 12 h. Migrated or invaded cells were photographed (left panel). Scale bars represent 500 µm. The bar graph on the right indicates the quantification of crystal violet stain eluted from the cell colonies or from migrated or invaded cells. Columns indicate mean (*n* = 3) ± SE * *p* < 0.05, ** *p* < 0.01, *** *p* < 0.001, **** *p* < 0.0001 statistically significant compared with normal cell line (MCW-OSE-1). (**C**) Representative images of three microscopic fields in a wound-healing assay of MCW-OV-SL3 cells with other indicated cell lines cells were captured at indicated time points. Red line indicates the empty space between healing cells. Scale bars represent 500 µm. (**D**) Protein lysates were prepared from indicated ovarian cancer cell lines and immunoblotted using indicated antibodies (see Figure S3). (**E**) Total RNA was isolated from indicated cell lines and qPCR was performed to determine the expression of the indicated genes. All mRNA expression was normalized to β-actin in qPCR assays. Histograms represent mean ± SE of triplicate determinations. Statistical analysis was undertaken using Student’s *t*-test. * *p* < 0.05, ** *p* < 0.01, *** *p* < 0.001, **** *p* < 0.0001, ns: non-significant.

First, we determined the proliferation rate of MCW-OV-SL-3 cells and MCW-OSE-1 cells, along with a set of ovarian cancer cells using manual cell counting and found that the doubling time of MCW-OV-SL-3 is similar to the growth rate of aggressively growing ovarian cancer cells and significantly higher than compared to the growth rate of normal ovarian surface epithelial cells MCW-OSE-1 (Figure 2A). We also performed the colony formation assay and 3D spheroid formation assay to determine the clonogenic potential of MCW-OV-SL-3 cells. Herein, we observed that MCW-OV-SL-3 and A2780 cells formed larger colonies and 3D spheroids as compared to normal ovarian surface epithelial cells MCW-OSE-1 and high grade serous ovarian cancer cell lines OVCAR-4 and OVCAR-8 in both 2D and 3D culture conditions. We also determined the migratory and invasive potential of the cell lines and found that the migration, invasion, and wound healing abilities of MCW-OV-SL-3 cells is significantly higher than MCW-OSE-1 and it was similar or higher than A2780 ovarian cancer cells (Figure 2B,C). We further validated the expression of genes which are critical for epithelial-to-mesenchymal transition (EMT) and cancer stemness by quantitative PCR (qPCR). Interestingly we found that expression of EMT-related genes such as N-Cadherin and vimentin was significantly high in MCW-OV-SL-3 and A2780 cell lines as compared to MCW-OSE-1, while E-Cadherin expression was significantly less in MCW-OV-SL-3 and A2780 cell lines. Moreover, expression of cancer stemness related genes such as CD44 and c-Kit was significantly high in MCW-OV-SL-3 and A2780 cell lines compared to normal ovarian surface epithelial cells MCW-OSE-1, while CD24 was significantly low in ovarian cancer cell lines MCW-OV-SL-3 and A2780 cells as compared to MCW-OSE-1 normal cells (Figure 2D,E).

### 3.4. MCW-OV-SL-3 Exhibited Spontaneous Transition from Monolayer to 3D Spheroid and from 3D Spheroid to Monolayer

**Figure 3:**
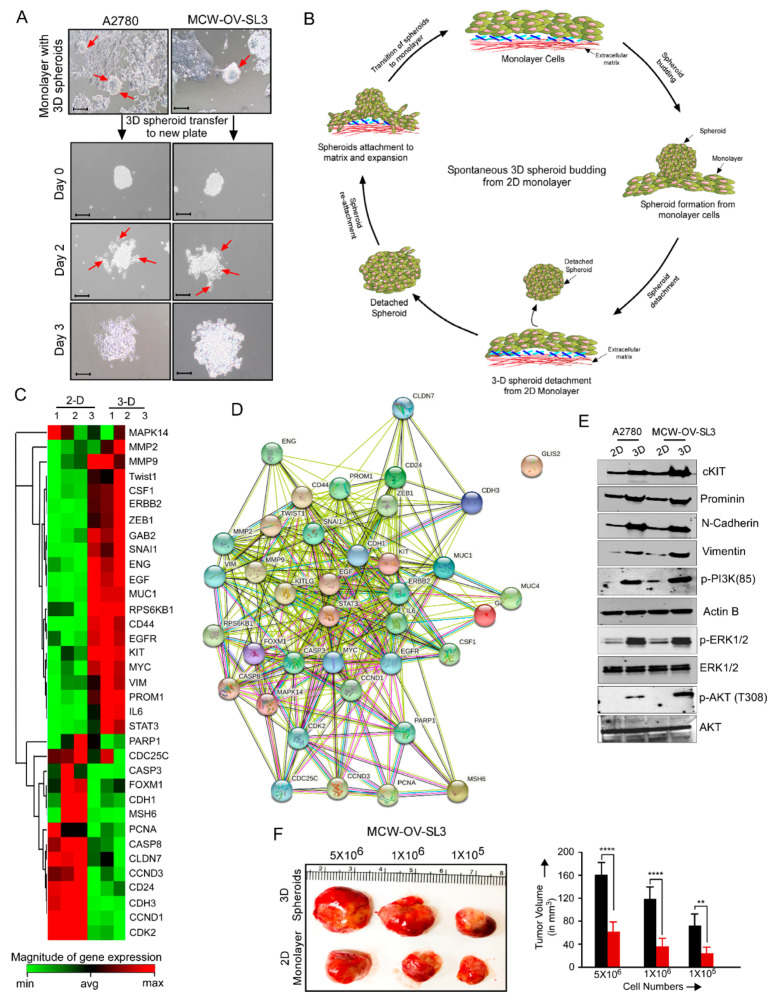
MCW-OV-SL3 cells form 3-D spheroids, which exhibit EMT and cancer stemness characteristics. (**A**) A2780 and MCW-OV-SL3 cells were grown as a monolayer in serum containing media and when tumor spheroids were detached from the monolayer, the spheroids were transferred to a new plate and phase contrast images were captured on days 0, 2, and 3. Red arrow denotes transition of 3D spheroids to 2D monolayer. (**B**) A schema showing the process of spontaneous 3D spheroid formation from a monolayer and its transition to monolayer cells. (**C**) Total RNA was prepared from 2D monolayer and 3D spheroids of MCW-OV-SL3 cells (*n* = 3) from ‘A’ and qPCR array was performed. Heatmap showing gene expression in fold change with a *p* value ≤ 0.05 was prepared using Qiagen RT-PCR profiler software. B2M, HPRT1, RPLP0, GAPDH, and ACTB genes were used as housekeeping genes for normalizing data. Gene expression ≥ 1.4-fold difference is indicated in red (upregulated) and ≤1.4-fold difference is indicated as green (downregulated) in the heatmap. (**D**) Proteins of up or downregulated genes from ‘C’ were analyzed for protein–protein interaction using String software. (**E**) Protein lysates were prepared from monolayer and tumor spheroids of A2780 and MCW-OV-SL3 ovarian cancer cell lines and immunoblotted using indicated antibodies (see Figure S3). (**F**) Indicated numbers of MCW-OV-SL-3 cells harvested from 2D monolayer culture or from 3D spheroids were injected subcutaneously on the left flank in female athymic nude mice (age: 4–6 weeks; *n* = 6/group). Tumors were isolated 6 weeks after inoculation, photographed, and tumor volume was quantified. ** *p* < 0.01, **** *p* < 0.0001, ns: non-significant.

We observed that both endometroid ovarian cancer cell lines, MCW-OV-SL-3 and A2780 cells formed spontaneously floating spheroids of ~30–100 µm diameter in size when the tumor cells became confluent within 2–3 days in the in vitro culture condition (Figure 3A). We also observed that the floating 3D spheroids form monolayers when transferred to a new culture dish and then formed floating spheroids consequently (Figure 3A,B).

To check the gene expression profile between the 2D monolayer and 3D spheroids population of the MCW-OV-SL-3 cell line, we performed a qPCR array to determine the change in the expression of genes which are important for cell proliferation, survival, EMT, and oncogenic signaling. We found that 3D spheroids of MCW-OV-SL-3 showed a high expression (red color) of the genes such as Twist1, ZEB1, SNAIL1, EGF, MUC1, EGFR, c-Kit, MYC, VIMENTIN, PROMININ, IL-6, and STAT3 compared to the adherent cells that are grown as a 2D monolayer form. Conversely, we found that FOXM1, PCNA, and Cyclin D1 are upregulated in the adherent cells as compared to the 3D spheroids (Figure 3C). Next, we determined the protein interaction network of these genes using String software and found a highly interactive protein network between cancer stemness and EMT related genes (Figure 3D). We further identified an increased phosphorylation of PI3K (p85), pAKT (T308), pAKT (S473), and pERK1/2 proteins in 3D spheroids as compared to the 2D monolayer cells. Next, we determined the tumor-initiating capacity of both 2D monolayer cells and 3D spheroids of MCW-OV-SL3 by injecting different numbers of cells (5 × 10^6^, 1 × 10^6^, and 1 × 10^5^ cells/mice) isolated from both monolayer 2D cells and 3D spheroids subcutaneously in the left flank region of nude mice. Here, we found that cancer cells from 3D spheroids form tumors with significantly increased size and volume as compared to the 2D monolayer condition in vivo (Figure 3F).

### 3.5. Cisplatin-Resistant MCW-OV-SL-3 Cells Exhibited Increased Phosphorylation of AKT and ERK Proteins and Tumor Growth

**Figure 4:**
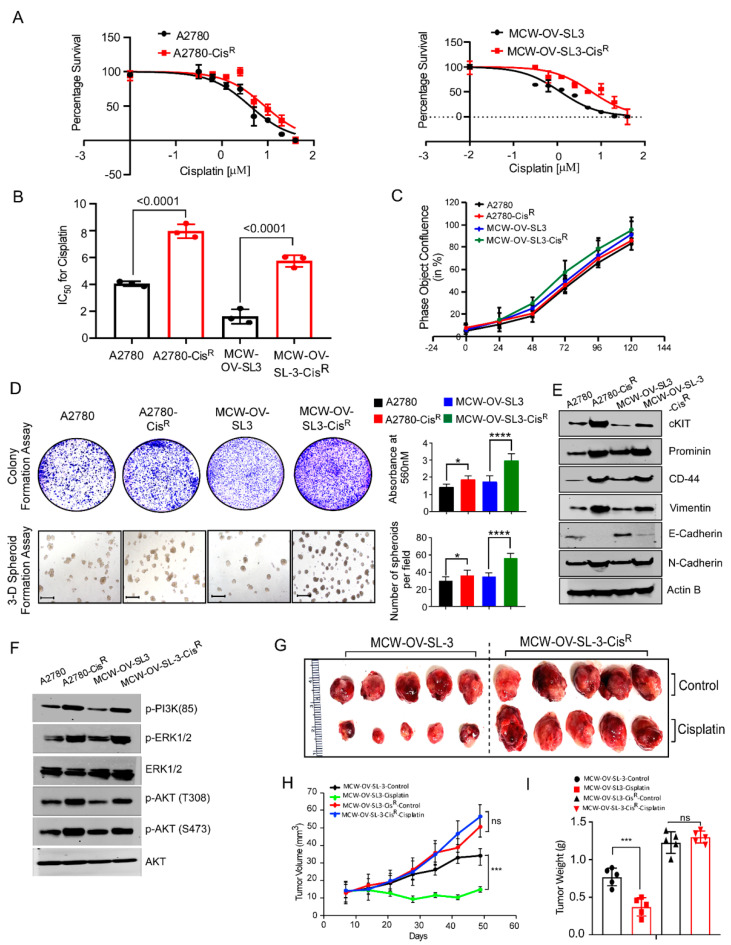
Cisplatin-resistant MCW-OV-SL3 cells exhibit hyperproliferation and aberrant PI3K/AKT activation. (**A**) Cisplatin-sensitive and resistant versions of A2780 and MCW-OV-SL-3 cells were grown in the presence of different concentrations of cisplatin for 48 h and then cell viability was determined using MTT. Error bars represent standard error of the mean. (**B**) IC50 of cisplatin-sensitive and resistant A2780 and MCW-OV-SL3 cells were determined from three independent cell viability experiments performed using MTT. (**C**) Cisplatin-sensitive and resistant of A2780 and MCW-OV-SL3 cells (3500 cells/well of each cell line) were seeded in 96-well culture plates and cell proliferation was assessed as percent confluency using an Incucyte S3 live-cell analysis system for a period of 120 h. (**D**) Cell lines (1000 cells/well) indicated were grown on six well culture plate for 14 days for colony formation, then stained using 0.5% crystal violet and photographed. For 3D formation assay, 3000 cells were grown on Matrigel-coated plates for seven days and then photographed using phase-contrast microscope. Scale bars represent 500 µm. The bar graph on right reveals the quantification of the colony formation and 3D spheroids of indicated cell lines. (**E**,**F**) Cell lysates were prepared from cisplatin-sensitive and resistant A2780 and MCW-OV-SL3 cells and immunoblot was performed with indicated antibodies. β-actin was used as a loading control (see Figure S3). (**G**) MCW-OV-SL3 and MCW-OV-SL3-CisR (1 × 10^6^ cells/mice) were injected subcutaneously in the left flank of 4 weeks old female athymic nude mice (*n* = 7/group). Mice were treated with 2.5 mg/kg body weight cisplatin or sterile PBS (vehicle control) once per week and tumors were isolated on day 42 after inoculation and photographed. Error bars represent SE of the mean. (**H**,**I**) Histograms represent tumor volume which was measured on the indicated days and tumor weight of the mice isolated in ‘G’ (*n* = 5/group). Error bar indicates SE of triplicate determination. ns-not significant, * *p* < 0.05, *** *p* < 0.001, **** *p* < 0.0001 statistically significant compared with control.

It is reported that aberrant activation of PI3K-AKT signaling causes chemoresistance [20,21,22]. To investigate this, we generated a cisplatin-resistant derivative of MCW-OV-SL3 after continuous exposure (more than 16 months) of increased cisplatin doses. Next, we compared cisplatin sensitivity in MCW-OV-SL3 cells with A2780, and A2780-CisR cell lines, and found that the IC50 of cisplatin in A2780 and A2780-CisR was 4.2 and 8.4 µM, respectively, and in MCW-OV-SL-3 and MCW-OV-SL-3-CisR was 2.2 and 6.2 µM, respectively (Figure 4A,B). We also checked the cell proliferation, clonogenic potential, and 3D spheroid formation ability of the parental and cisplatin-resistant derivative of both A2780 and MCW-OV-SL3 cells. Interestingly we found that the cisplatin-resistant (CisR) version of both cell lines exhibited high clonogenic potential and 3D spheroid formation ability (Figure 4C,D) as compared to parental A2780 and MCW-OV-SL3. We next determined the levels of EMT markers (E-Cadherin, N-Cadherin) and cancer stemness-related markers (c-Kit, CD44, and prominin) in both cell lines and found that the cisplatin-resistant derivative of cell lines expressed high levels of N-Cadherin and low levels of E-Cadherin as well as c-Kit, CD44, and prominin proteins (Figure 4E). Since we observed an aberrant activation of PI3K-AKT signaling in MCW-OV-SL-3 cells, we investigated whether there was any further increase in the PI3K-AKT signaling in the cisplatin-resistant cell lines. Herein, we found that both the cisplatin-resistant version of the cell lines constitutively expressed the phosphorylated form of p-PI3K (85), pERK1/2, Akt (T308), and Akt (Ser473) (Figure 4F). We further studied the tumor growth potential of both the parental and cisplatin-resistant derivatives of both MCW-OV-SL3 and MCW-OV-SL3-CisR cells in vivo, and the mice were subsequently treated with cisplatin or vehicle control in accordance with protocol approved by the Institutional Animal Care and Use Committee (IACUC) at the Medical College of Wisconsin. Herein, we noticed that cisplatin treatment significantly reduced the tumor volume and weight of cisplatin-sensitive MCW-OV-SL-3 cells but not the MCW-OV-SL-3-CisR cells (Figure 4G–I).

### 3.6. PI3K/Akt Signaling Inhibition Sensitized the Cisplatin Resistance and Inhibits Tumorigenic Properties in Ovarian Cancer

**Figure 5:**
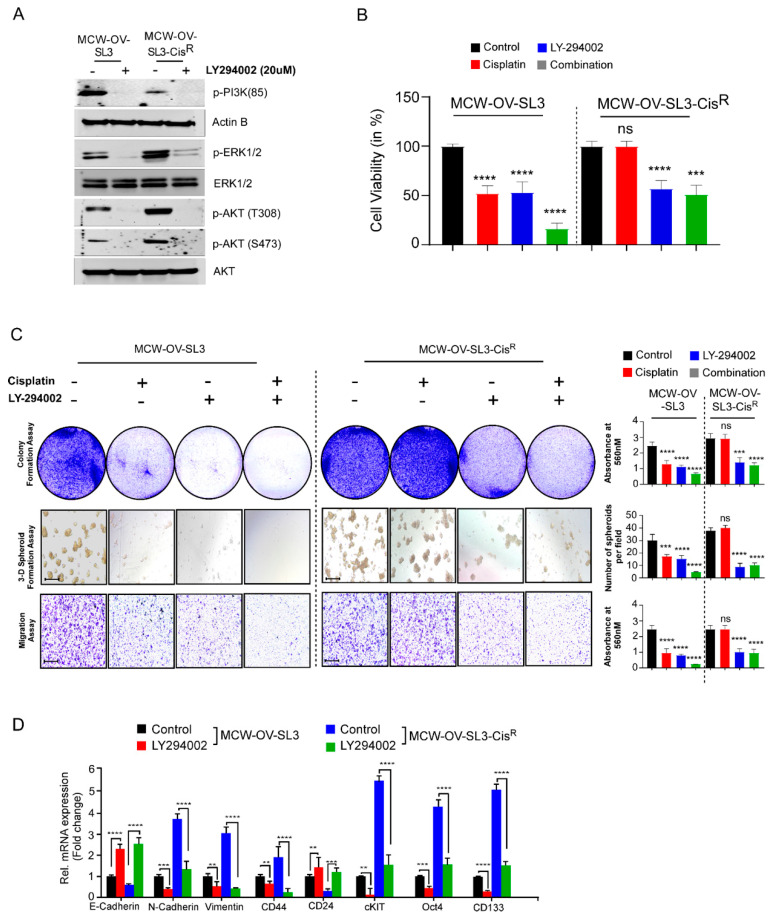
PI3K inhibitor treatment reduced EMT, cancer stemness, and chemoresistance in the MCW-OV-SL3 cell line. (**A**) MCW-OV-SL3 and MCW-OV-SL3-CisR cells were treated for 24 h with LY-294002. Cell lysates were prepared and immunoblotted using the antibodies indicated (see Figure S3). (**B**) MCW-OV-SL3 and MCW-OV-SL3-CisR cells were treated with either cisplatin (2.2 and 6.2 µM), LY-294002 (5 and 10 µM), or their combination (cisplatin 1.1 and 3.1 µM), LY-294002 (2.5 and 5 µM) for 48 h and the cell viability was assessed by MTT assay at 48 h. Results are representative of three independent experiments performed in triplicates and error bars represent SE of the mean. (**C**) MCW-OV-SL-3 and MCW-OV-SL-3-CisR cell lines treated for 48 h with either cisplatin, LY294002, or in combination (as treated in **B**) were grown on 6-well culture plates for 14 days for colony formation, then stained using 0.5% crystal violet and photographed. For 3D formation assay, cells were grown on Matrigel-coated plates for seven days and then photographed using a phase-contrast microscope. For cell migration assay, cells were plated on trans-well inserts without Matrigel for 12 h. Migrated cells were photographed (left panel). Scale bars represent 500 µm. The right bar graph reveals the quantification of the colony formation, 3D spheroids, and migratory ability of indicated cell lines. (**D**) Total RNA was isolated, and qPCR was performed from MCW-OV-SL-3 and MCW-OV-SL-3-CisR cell lines treated with either cisplatin, LY294002, or in combination (as treated in **B**) for 48 h. mRNA expression was normalized to GAPDH. Bars represent SE of triplicate determinations. Error bar indicates SE of triplicate determination. ns-not significant, **p < 0.01, *** *p* < 0.001, **** *p* < 0.0001 statistically significant compared with control.

Next, we determined the effect of blocking PI3K-AKT signaling by PI3K inhibitor LY294002 and confirmed that inhibition of PI3K and AKT phosphorylation (Figure 5A and Figure S2A), inhibited the cell viability, colony formation, cell migration, and 3D morphogenesis in both parental and cisplatin-resistant cells. Conversely, we observed that cisplatin treatment reduced the cell viability, colony formation, cell migration, and 3D morphogenesis of parental MCW-OV-SL3 or A2780 cells but not the cisplatin-resistant MCW-OV-SL3 or A2780 cells (Figure 5B,C and Figure S2B,C). We further observed that LY294002 treatment reduced EMT by increasing the level of E-cadherin and decreasing the level of N-Cadherin, vimentin, and the markers of cancer stemness, where LY294 decreased the level of c-Kit, Oct4, CD133, and increased the level of CD24 in both cisplatin-sensitive and cisplatin-resistant versions of A2780 and MCW-OV-SL-3 cell lines (Figure 5D and Figure S2D).

## 4. Discussion

Dissecting the genomic alterations and transcriptomic landscapes of well characterized cell lines of a histological subtype will allow for the study of the molecular mechanisms underlying the progression and chemoresistance in ovarian cancer. In this regard, we established an endometrioid ovarian cancer cell line that forms tumor spheroids when confluent as floating spheroids, which occurs in ovarian cancer patients during metastasis. Recently, we demonstrated a mechanism that underlies the transition from a monolayer of adherent cells to non-adherent 3D spheroids, where we found that in HGSOC cell lines the adherent cells are highly proliferative and rely on the FOXM1 transcription factor for cell adhesion and proliferation [23]. In contrast, we also found that the non-adherent spheroids in HGSOC cell lines rely on EGFR or ERBB2 signaling for their survival and growth [23]. However, the exact mechanism that facilitates spheroid formation in conjunction with cancer stemness and chemoresistance is poorly studied in the endometrioid subtype of ovarian cancer. Therefore, we established an MCW-OV-SL3 cell line to characterize the underlying mechanism in endometrioid ovarian cancer and determined the role of chromosomal alteration in chemoresistance and cancer cell aggressiveness.

Chromosomal abnormalities are common phenomena in development and progression of many human cancers including ovarian carcinoma [24]. Previously, we have demonstrated that several genes including both protein coding and non-coding genes are aberrantly expressed due to copy number gain in ovarian cancer and in breast cancer [17,24,25,26,27,28]. Moreover, chromosomal abnormalities in 1q21 such as amplifications, rearrangements, and translocations, have been reported in several hematological malignancies and solid tumors including endometroid ovarian carcinoma [29,30,31]. In conjunction, we identified that chromosomal abnormalities in the 1q (q21–q42) locus in MCW-OV-SL3 are an important contributor to cancer stemness in highly aggressive tumors. Importantly, the interstitial duplication in the 1q21–q42 locus resulted in the activation of PI3K/AKT signaling, which is likely to be a major reason for chemoresistance. Thus, the genes located in the 1q21–q42 locus could serve as reliable indicators to predict the out-come of chemotherapy as well as the need for PI3K inhibitors for cancer therapy. We also observed that PIK3C2, PIP5K1B, and AKT3 are highly frequently amplified in the TCGA dataset of ovarian cancer.

Among the genes located in 1q (21–42) are protein kinase B (AKT 1–3), PIK3CA, and PTEN, which are frequently hyperactivated or deleted in nearly 12–20% of patients with endometroid ovarian carcinoma [32]. It is well known that PIK3C2, PIP5K1B, AKT3 are either amplified or mutated in many cancers including ovarian cancer [2]. The PIK3CA gene encodes the catalytic subunit of phosphatidylinositol 3-kinase (PI3K-p110α), and AKT is the downstream effector in the PI3K pathway. PI3K proteins are heterodimers composed of a catalytic p110 subunit (PIK3CA) and a regulatory p85 subunit (PIK3R) that mediate receptor binding and activation. PI3K directly binds to phosphotyrosine residues of growth factor receptors or adaptors through pleckstrin homology domains leading to allosteric activation of the catalytic p110 subunit, and the phosphorylation of phosphatidylinositol 4,5-bisphosphate (PIP2) converts it to the active second messenger, PIP3. As a result, the PI3K complex is recruited to the plasma membrane and activates the pyruvate dehydrogenase kinase 1 (PDK1) and Akt proteins [33]. The PI3K/AKT pathway plays a significant role in the pathogenesis of ovarian cancer growth, survival, metabolic programing, autophagy, transcription regulation, and angiogenesis [34,35,36].

We noticed that MCW-OV-SL-3 and A2780 cells exhibited similar signaling mechanisms and dependency on PI3K/Akt signaling for spheroid formation and cancer stemness. It has also been observed that these cells do not adhere firmly to plastic and glass surfaces as is common with other cell lines and can be detached easily unlike other ovarian cancer cell lines, suggesting that these are aggressive cell lines and likely to be highly metastatic through peritoneal spreading, which is a hallmark of ovarian cancer metastasis [12].

In this study, we observed that a hyperactive PI3K/Akt cascade is associated with the spheroid formation, cancer stemness, chemoresistance, and epithelial to mesenchymal transition in the MCW-OV-SL3 cell line, and aggressive growth of the cisplatin-resistant versions of MCW-OV-SL3 cells. The mechanism of chemoresistance is multifactorial and is proposed to arise from a physical barrier to drug penetration, induction of genes or signaling pathways that enhance survival of drug-resistant cancer stem cell subpopulations, or due to chromosomal alterations and epigenetic disposition [37,38]. In this study, we found that the treatment of parental and cisplatin-resistant MCW-OV-SL3 with PI3K-Akt inhibitor LY294002 decreased PI3K/AKT/MEK signaling. We also found that LY294002 inhibitor decreased cell viability, migration, invasion, and spheroid forming ability of chemo-resistant MCW-OV-SL3-CisR. Taken together, our data support the notion that the cell line we developed is clinically relevant to study the pathophysiology and drug resistance mechanism in ovarian cancer. Studies like ours that use the primary cell lines established from patient samples for testing of chromosomal aberrations and the associated mechanism of tumor progression and chemoresistance through tumor spheroid formation ability will identify appropriate regiments for personalized therapy for ovarian cancer.

## 5. Conclusions

In this study we established an endometrioid subtype of ovarian cancer cell line, MCW-OV-SL-3, which is tumorigenic and highly metastatic like A-2780 ovarian cancer cell line. We observed this cell line has interstitial duplication of 1q(q21-q42) chromosome locus. Consequently, this duplication resulted into high PIK3C2B expression and aberrant PI3K-AKT-ERK signaling. We also observed that aberrant PI3K-AKT-ERK signaling promoted cancer stemness characteristics, chemoresistance and EMT. In conjunction, the inhibition of PI3K-AKT-ERK signaling by a PI3K-AKT dual kinase inhibitor abrogated the oncogenic characteristics such as cancer stemness, chemoresistance and EMT. Taken together, our study suggested that copy number aberration of 1q locus is a critical driver in endometrioid subtype ovarian cancer through PI3K signaling.

## Data Availability

The data presented in this study are available on request from the corresponding author.

## Supplementary Materials

The following supporting information can be downloaded at: https://www.mdpi.com/2072-6694/14/4/958/s1. Figure S1: Genetic alterations and expression changes in PI3K pathway components in HGSOC patient datasets, Figure S2: Inhibition of PI3K-AKT signaling axis by LY-294002 (PI3K inhibitor) decreases EMT expression, cancer stemness, and chemoresistance, Figure S3: Full Western blot figures, Table S1: List of primers used, Table S2: List and sources of antibodies used.
