# Correction: Parashar et al. Patient-Derived Ovarian Cancer Spheroids Rely on PI3K-AKT Signaling Addiction for Cancer Stemness and Chemoresistance. *Cancers* 2022, *14*, 958

**DOI:** 10.3390/cancers14102443

**Published:** 2022-05-16

**Authors:** Deepak Parashar, Anjali Geethadevi, Sonam Mittal, Lindsey A. McAlarnen, Jasmine George, Ishaque P. Kadamberi, Prachi Gupta, Denise S. Uyar, Elizabeth E. Hopp, Holli Drendel, Erin A. Bishop, William H. Bradley, Kathleen M. Bone, Janet S. Rader, Sunila Pradeep, Pradeep Chaluvally-Raghavan

**Affiliations:** 1 Department of Obstetrics and Gynecology, Medical College of Wisconsin, Milwaukee, WI 53226, USA; dparashar@mcw.edu (D.P.); ageethadevi@mcw.edu (A.G.); smittal@mcw.edu (S.M.); lmcalarnen@mcw.edu (L.A.M.); jasgeorge@mcw.edu (J.G.); ipulikkal@mcw.edu (I.P.K.); prgupta@mcw.edu (P.G.); duyar@mcw.edu (D.S.U.); ehopp@mcw.edu (E.E.H.); erbishop@mcw.edu (E.A.B.); wbradley@mcw.edu (W.H.B.); jrader@mcw.edu (J.S.R.); spradeep@mcw.edu (S.P.); 2 Department of Pathology, Medical College of Wisconsin, Milwaukee, WI 53226, USA; hdrendel@mcw.edu (H.D.); kbone@mcw.edu (K.M.B.); 3 Department of Physiology, Medical College of Wisconsin, Milwaukee, WI 53226, USA; 4 Cancer Center, Medical College of Wisconsin, Milwaukee, WI 53226, USA

The authors would like to correct the author byline to include Dr. Kathleen M. Bone, whose earned authorship was inadvertently omitted from the original submission of the published paper [1]. Dr. Bone should be listed as the 13th author, between William H. Bradley and Janet S. Rader, and be affiliated with the “Department of Pathology, Medical College of Wisconsin” (affiliation number 2).

Dr. Bone has clearly earned authorship on this study for the following contributions, because she:

(1) Oversaw, reviewed, analyzed, interpreted, and validated karyotyping of the two cell lines (MCW-OV-SL-3 and MCW-OSE-1) in the Cytogenetics Laboratory.
(2) Helped write up description of methods and results of karyotyping used for Figure 1, panel C and Figure S1, panel B (specifically Section 2.5).

All authors, including the senior author, Dr. Pradeep Chaluvally-Raghavan, agree with the inclusion of Dr. Kathleen M. Bone as a co-author on this publication. 

The “Author Contribution” statement should also be updated to the following version:

**Author Contributions:** Conceptualization, D.P., S.P. and P.C.-R.; Funding acquisition and supervision, S.P. and P.C.-R.; Experimental plan, D.P., S.P. and P.C.-R.; Methodology and investigation, D.P., A.G., S.M., S.P. and P.C.-R.; In vivo and in vitro experiments, D.P., A.G. and S.P.; Clinical sample collection and data analysis, L.A.M., D.S.U., W.H.B., E.E.H., E.A.B. and J.S.R.; Data analysis, D.P., A.G., S.P. and P.C.-R.; Karyotyping, H.D. and K.M.B.; Figure preparation, D.P. and P.C.-R.; Writing—original draft, D.P., A.G., S.M., S.P. and P.C.-R.; Writing—review and editing, D.P., A.G., S.M., L.A.M., J.G., I.P.K., P.G., S.P. and P.C.-R.; Funding allocation, P.C.-R. and S.P. All authors have read and agreed to the published version of the manuscript.

The authors apologize for any inconvenience caused and state that the scientific conclusions are unaffected. The original article has been updated.
