# Supplementary material for: Patient-Derived Ovarian Cancer Spheroids Rely on PI3K-AKT Signaling Addiction for Cancer Stemness and Chemoresistance
Source: Cancers (Basel). 2022 Feb 15;14(4):958. doi: 10.3390/cancers14040958 (PMC8870411; doi:10.3390/cancers14040958)
Supplement: Supplementary file 1 [file cancers-14-00958-s001.zip › cancers-1540951-supplementary.pdf]

A

MCW-OV-SL-3

Cell Check Analysis

Species -specific PCR Evaluation	
Species	MCW-OV-SL-3
Mouse	-
Rat	-
Human	+
Chinese hamster	-
African green monkey	-

STR Marker Analysis

Marker Name	Sample Results	MCW-OV-SL-3
AMEL	X	NA
CSF1PO	10, 11	NA
D13S317	12, 13	NA
D16S539	11, 13	NA
D18S51	16, 18	NA
D21S11	28	NA
D3S1358	14, 16	NA
D5S818	11,12	NA
D7S820	10	NA
D8S1179	15, 17	NA
FGA	19, 24	NA
Penta_D	8, 9	NA
Penta_E	10, 14	NA
TH01	6	NA
TPOX	8, 10	NA
vWA	15, 16	NA
Identity Match	N/A	N/A

B MCW-OSE-1

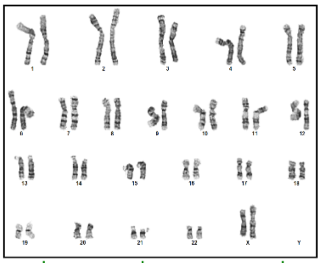

C

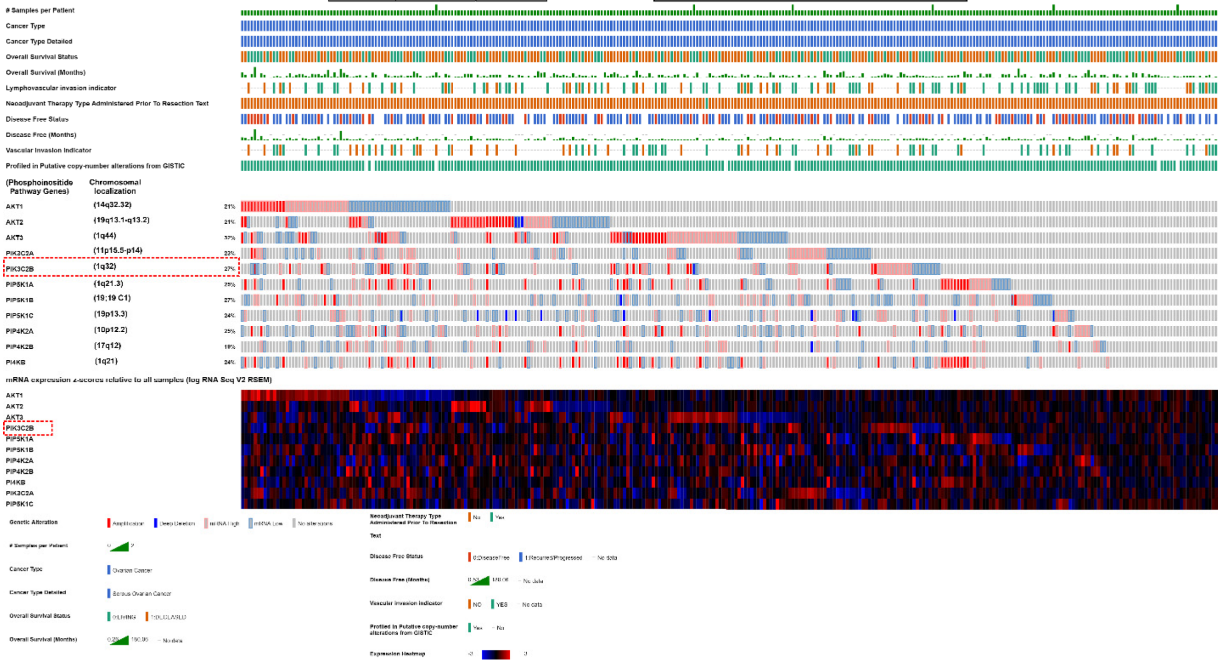

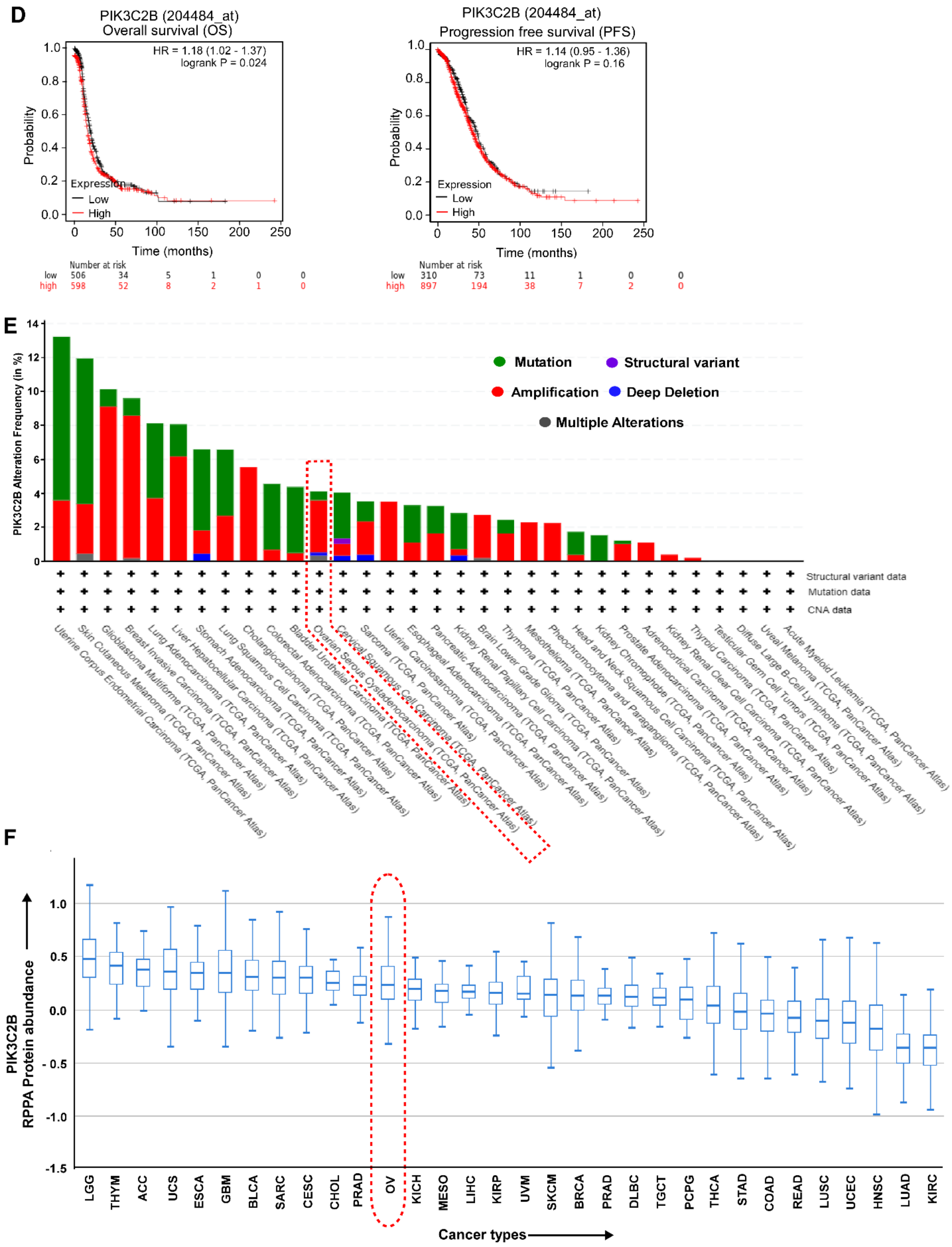

D
PIK3C2B (204484_at)
Overall survival (OS)
HR = 1.18 (1.02 - 1.37)
logrank P = 0.024
Probability
Time (months)
Expression
Low
High
Number at risk
low 506 34 5 1 0 0
high 598 52 8 2 1 0
PIK3C2B (204484_at)
Progression free survival (PFS)
HR = 1.14 (0.95 - 1.36)
logrank P = 0.16
Probability
Time (months)
Expression
Low
High
Number at risk
low 310 73 11 1 0 0
high 897 194 38 7 2 0
E
PIK3C2B Alteration Frequency (in %)
Mutation
Structural variant
Amplification
Deep Deletion
Multiple Alterations
Structural variant data
Mutation data
CNA data
Uterine Corpus Endometrial Carcinoma (TCGA, PanCancer Atlas)
Skin Cutaneous Melanoma (TCGA, PanCancer Atlas)
Glioblastoma Multiforme (TCGA, PanCancer Atlas)
Breast Invasive Carcinoma (TCGA, PanCancer Atlas)
Lung Adenocarcinoma (TCGA, PanCancer Atlas)
Liver Hepatocellular Carcinoma (TCGA, PanCancer Atlas)
Stomach Adenocarcinoma (TCGA, PanCancer Atlas)
Lung Squamous Cell Carcinoma (TCGA, PanCancer Atlas)
Cholangiocarcinoma (TCGA, PanCancer Atlas)
Colorectal Adenocarcinoma (TCGA, PanCancer Atlas)
Bladder Urothelial Carcinoma (TCGA, PanCancer Atlas)
Ovarian Serous Cystadenocarcinoma (TCGA, PanCancer Atlas)
Cervical Squamous Cell Carcinoma (TCGA, PanCancer Atlas)
Sarcoma (TCGA, PanCancer Atlas)
Uterine Carcinosarcoma (TCGA, PanCancer Atlas)
Esophageal Adenocarcinoma (TCGA, PanCancer Atlas)
Pancreatic Adenocarcinoma (TCGA, PanCancer Atlas)
Kidney Renal Papillary Cell Carcinoma (TCGA, PanCancer Atlas)
Brain Lower Grade Glioma (TCGA, PanCancer Atlas)
Thymoma (TCGA, PanCancer Atlas)
Mesothelioma (TCGA, PanCancer Atlas)
Pheochromocytoma and Paraganglioma (TCGA, PanCancer Atlas)
Head and Neck Squamous Cell Carcinoma (TCGA, PanCancer Atlas)
Kidney Chromophobe (TCGA, PanCancer Atlas)
Prostate Adenocarcinoma (TCGA, PanCancer Atlas)
Adrenocortical Carcinoma (TCGA, PanCancer Atlas)
Kidney Renal Clear Cell Carcinoma (TCGA, PanCancer Atlas)
Thyroid Carcinoma (TCGA, PanCancer Atlas)
Testicular Germ Cell Tumors (TCGA, PanCancer Atlas)
Diffuse Large B-Cell Lymphoma (TCGA, PanCancer Atlas)
Uveal Melanoma (TCGA, PanCancer Atlas)
Acute Myeloid Leukemia (TCGA, PanCancer Atlas)
F
PIK3C2B
RPPA Protein abundance
LGG THYM ACC UCS ESCA GBM BLCA SARC CESC CHOL PRAD OV KICH MESO LIHC KIRP UVM SKCM BRCA PRAD DLBC TGCT PCPG THCA STAD COAD READ LUSC UCEC HNSC LUAD KIRC
Cancer types

G

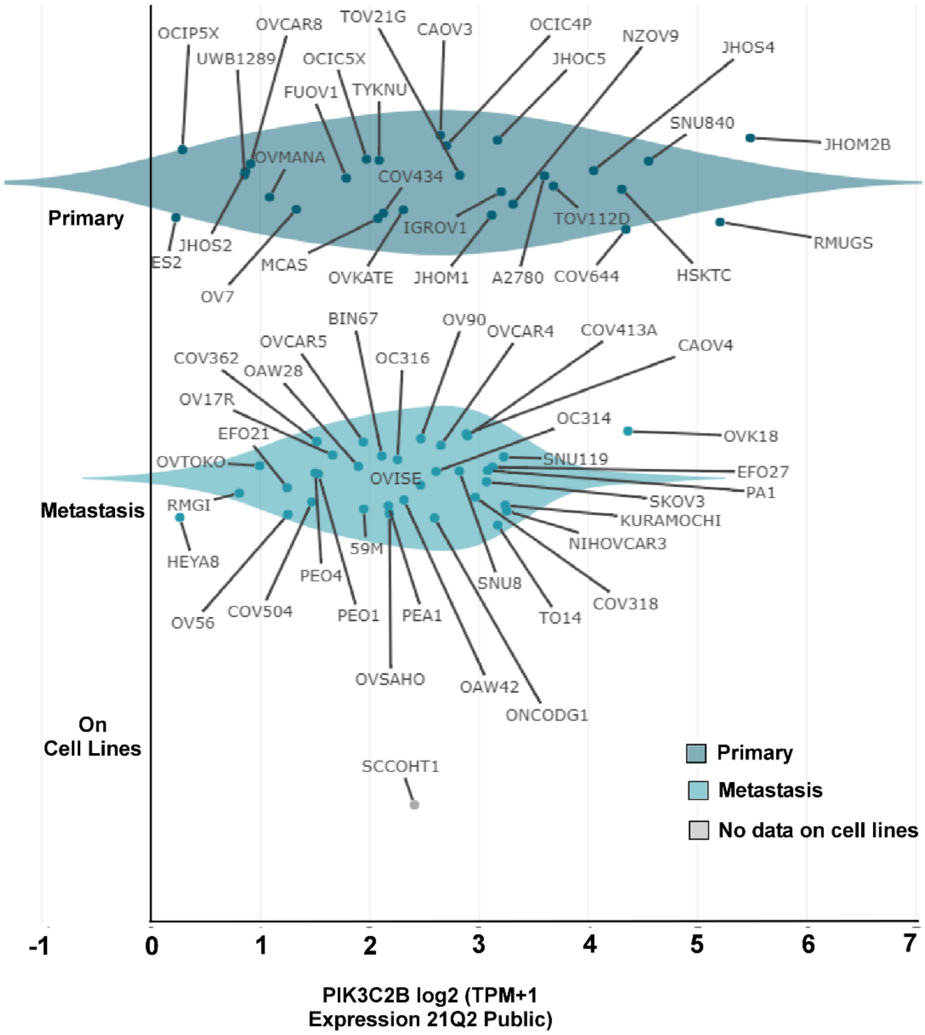

Figure S1 (Related to main figure 1) Genomic alterations and expression of PI3K pathway components in TCGA ovarian cancer data. (A).A panel of biomarkers used for STR profiling to determine cell line identity of MCW-OV-SL3 cells. (B) Representative karyogram image of MCW-OSE-1 cells. (C) Genetic alterations and expression profile changes of PIK3C2B pathway components in HGSOC patients data obtained from TCGA data. Copy number and expression profile was generated using cBioPortal (http://www.cbioportal.org accessed on 8 April 2021). (D). Overall survival and progression free survival analysis in high grade serous ovarian cancer patients, whose samples were stratified based on high versus low expression of PIK3C2B expression using KM survival analysis plotter (https://kmplot.com/analysis/ accessed on 8 April 2021). (E). Percentage frequency of different genetic and transcriptional alterations of PIK3C2B in various cancer tissues. (F). PIK3C2B protein abundance in various cancer tissues were determined in the Cancer Proteome Atlas datasets (https://tcpaportal.org/tcpa/ accessed on 8 April 2021). Red dotted lines indicate abundance of PIK3C2B in ovarian cancer tissues. (G) PIK3C2B mRNA expression was determined in ovarian cancer cell lines DepMap (https://depmap.org/portal/ accessed on 8 April 2021) and represented as log2 fold change analyzed using.

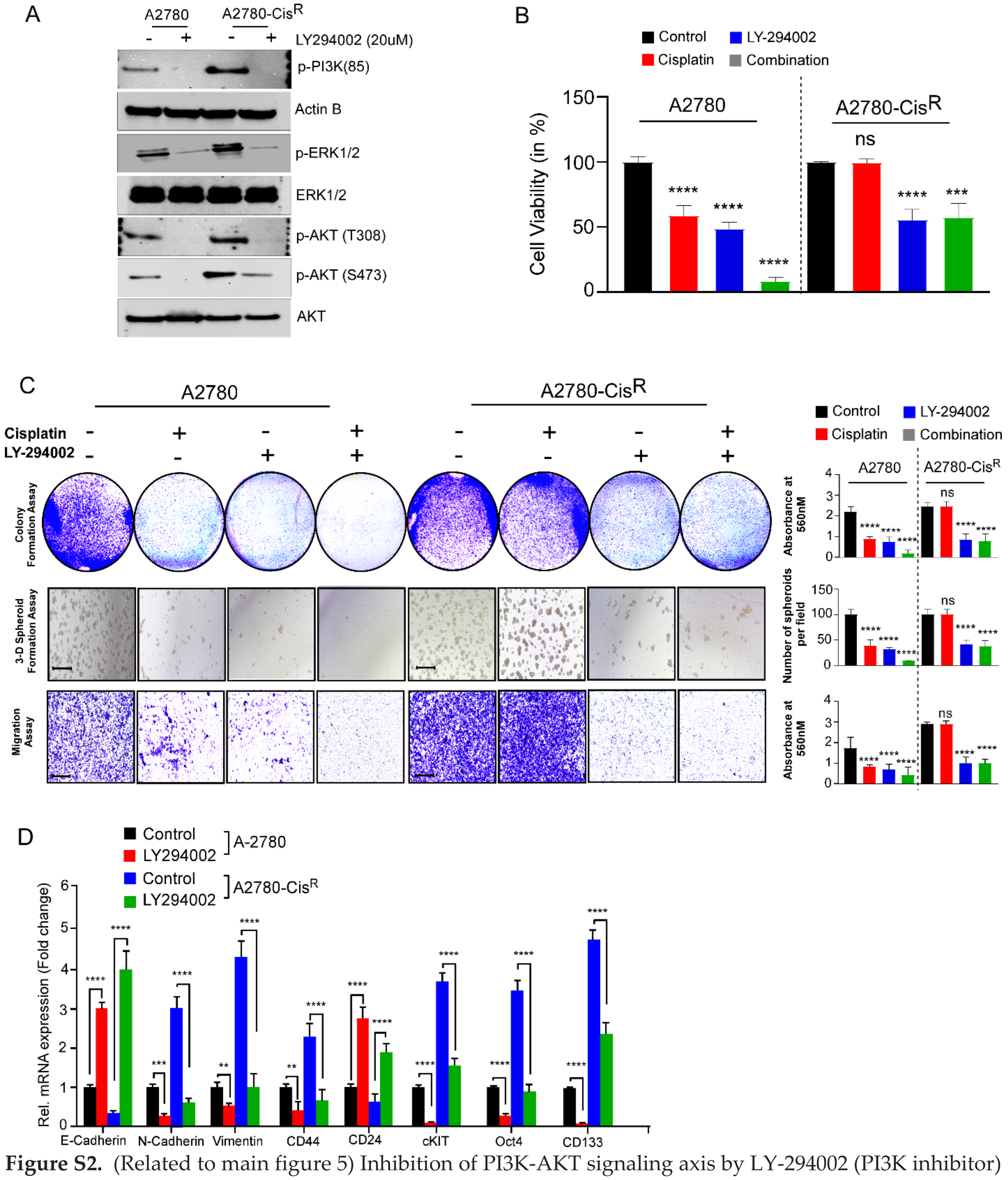

Figure S2. (Related to main figure 5) Inhibition of PI3K-AKT signaling axis by LY-294002 (PI3K inhibitor) significantly decrease EMT expression, cancer stemness and chemoresistance in A2780 cell line. (A). A2780 and A2780-CisR cells were treated for 48 h with either cisplatin, LY-294002 or their combination for 48 h. Cell lysates were prepared and immunoblotted using the antibodies indicated (see Figure-S3). (B). A2780 and A2780-CisR cells were treated with either cisplatin, LY-294002 or in combination for 48 h and the cell viability was assessed by MTT assay at 48h. Results are representative of three independent experiments performed in triplicates and error bars represent SE of the mean.(C). A2780 and A2780-CisR cell lines were

treated for 48 h with either cisplatin, LY294002 or in combination for 14 days for assessment of colony formation, then stained using 0.5% crystal violet and photographed. Stained colonies were dissolved and quantitated by measuring absorbance at 560nm and quantitated (right panel). For 3D formation assay, cells were grown on matrigel coated plate for seven days and then photographed using phase-contrast microscope (scale bars represent 500 μm). Number of spheroid colonies were counted from three separate fields and quantitated (right panel). For cell migration assay, cells were plated on trans-well inserts without Matrigel for 12 h. Migrated cells were photographed (left panel). Scale bars represent 500 μm and stained cells were dissolved using acetic acid and quantitated (right panel). Error bar indicates SE of triplicate determination. ** $p < 0.01$, ***; $p < 0.001$, ****; $p < 0.0001$ statistically significant compared with respective controls. (D). Total RNA was isolated, and qPCR was performed from A2780 and A2780-CisR cell lines treated with either cisplatin or LY294002. mRNA expression was normalized to GAPDH. Bars represent SE of triplicate determinations.

Related to main figure 1

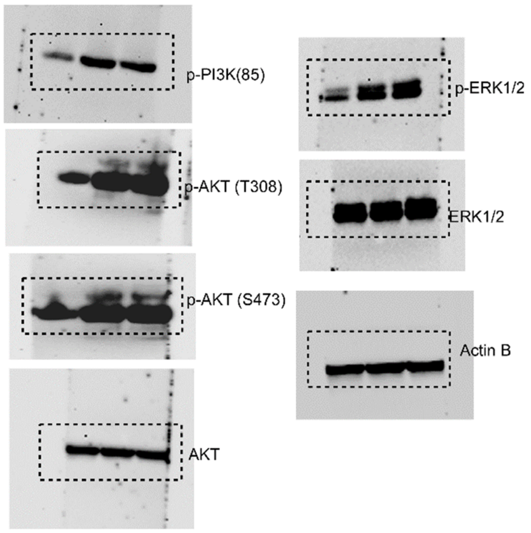

Related to main figure 2

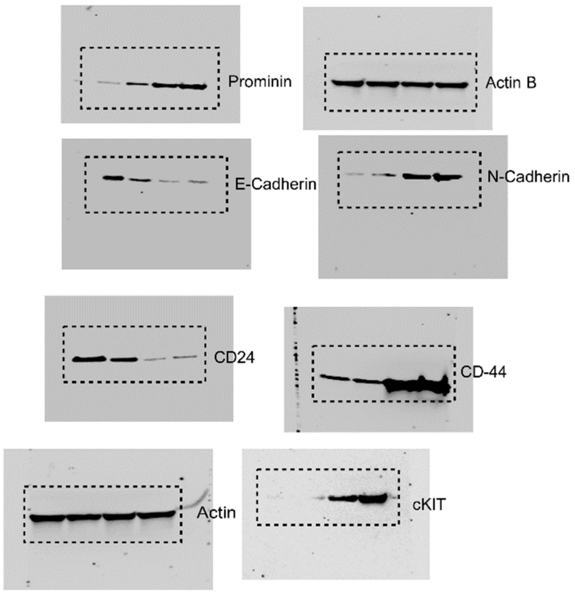

Related to main figure 3

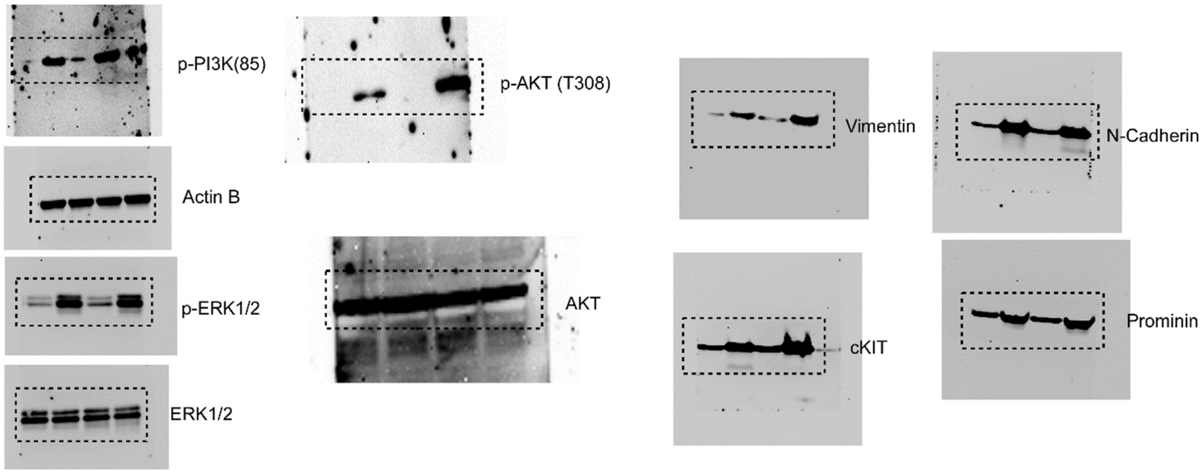

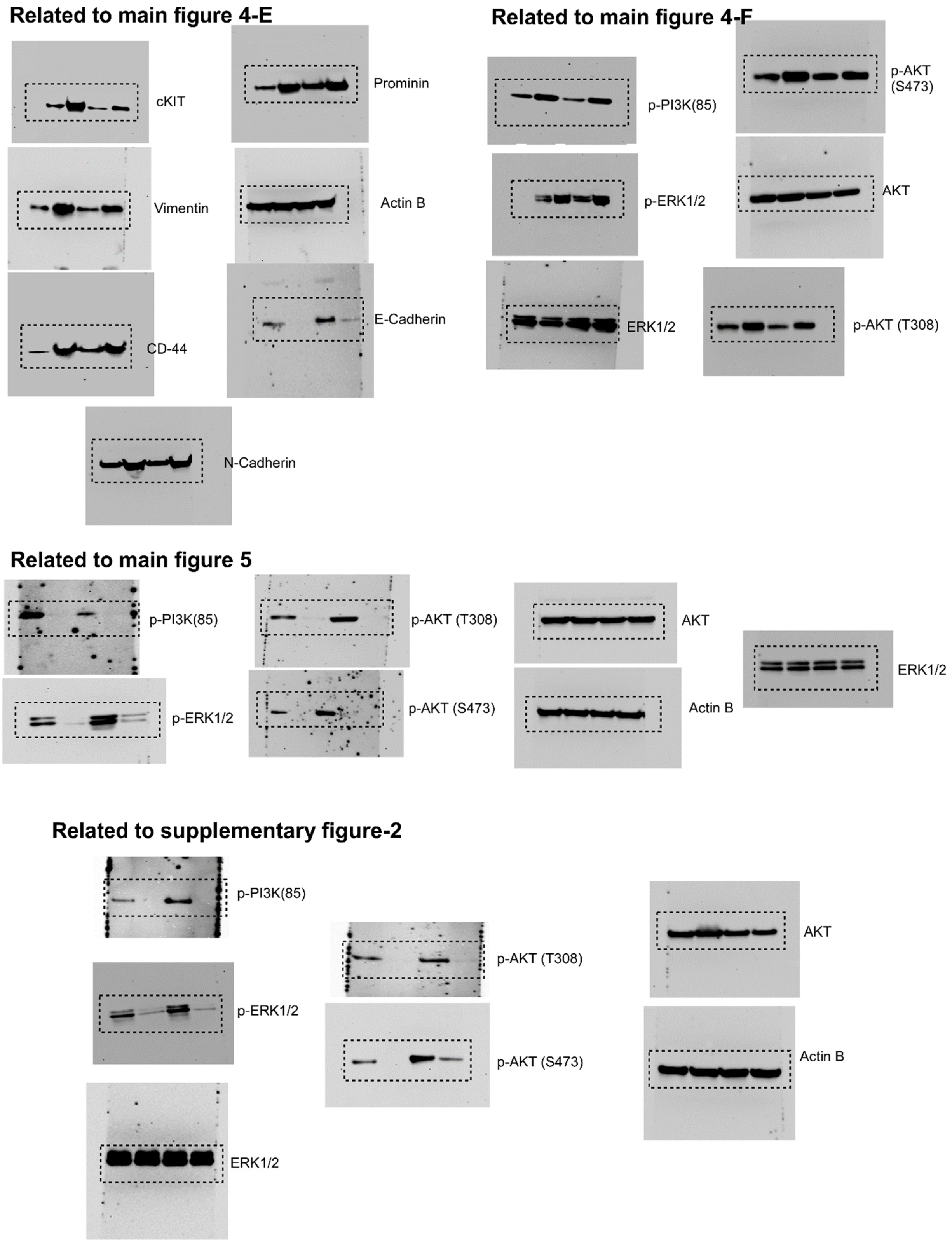

Figure S3. Full western blot Figures

Table S1. related to method section of quantitative real-time PCR
List of Primers were used for qPCR assays:

**Gene primers**	**Direction**	**Primer sequence**
E- Cadherin	Forward	GCT GGA CCG AGA GAG TTT CC
E- Cadherin	Reverse	CAA AAT CCA AGC CCG TGG TG
N-Cadherin	Forward	TGA CAA TGA CCC CAC AGC TC
N-Cadherin	Reverse	GTC CTG CTC ACC ACC ACT AC
Vimentin	Forward	GCA GGA GGC AGA AGA ATG GT
Vimentin	Reverse	CCA CTT CAC AGG TGA GGG AC
CD44	Forward	CCT GGC AGC CCC GAT TAT TT
CD44	Reverse	AAG GAC ACA CCC AAG CAA GG
CD24	Forward	GAG AGA TAA CCC TGC CCG AG
CD24	Reverse	CAA AAG AAA AGT CCG CGC CT
CD117	Forward	GCA CAA TGG CAC GGT TGA AT
CD117	Reverse	GGT GTG GGG ATG GAT TTG CT
OCT4	Forward	GAG AGG CAA CCT GGA GAATTT
OCT4	Reverse	ACT CGG ACC ACA TCC TTC T
ACTB	Forward	GTC ATT CCA AAT ATG AGA TGC GT
ACTB	Reverse	GCT ATC ACC TCC CCT GTG TG

Table-S2, REAGENT or RESOURCE SOURCE IDENTIFIER

**Antibodies:**		
Anti-cKIT	Cell Signaling Technology	Cat# 3074, RRID: AB_1147633
Anti-Vimentin	Abcam	Cat# ab76601; RRID: AB_1566863
Anti-Prominin	Cell Signaling Technology	Cat# 64326, RRID: AB_2721172
Anti-Beta Actin	Santa Cruz Biotechnology	Cat# sc-58679; RRID: AB_772478
Anti-p-PI3K (85)	Cell Signaling Technology	Cat# 4257; RRID: AB_659889
Anti-pERK1/2	Cell Signaling Technology	Cat# 5726; RRID: AB_2797617
Anti-ERK1/2	Cell Signaling Technology	Cat# 9108; RRID: AB_2141156
Anti-pAKT (T308)	Cell Signaling Technology	Cat #13038; RRID: AB_2629447
Anti-pAKT (S473)	Cell Signaling Technology	Cat# 4060, RRID: AB_2315049
Anti-AKT	Cell Signaling Technology	Cat# 2920; RRID: AB_1147620
Anti-rabbit IgG, HRP-linked Antibody	Cell Signaling Technology	Cat# 7074, RRID: AB_2099233
Anti-mouse IgG, HRP-linked Antibody	Cell Signaling Technology	Cat# 7076, RRID: AB_330924
**Chemicals:**		
DMEM	Thermo Fisher Scientific	Cat# 10569010
DMEM/F12 1:1	Thermo Fisher Scientific	Cat# 10565018
FBS	Atlanta Biologicals	Cat# H17112
Antibiotic (Penicillin/Streptomycin)	Thermo Fisher Scientific	Cat# 15140122
DMSO	Sigma Aldrich	Cat# D8418
Trypan Blue	Sigma Aldrich	Cat# T8154
Glutaraldehyde	Sigma Aldrich	Cat# G7651
Crystal Violet	Sigma Aldrich	Cat# C6158
Acetic acid	Sigma Aldrich	Cat# A6283
Trypsin	Thermo Fisher Scientific	Cat# 15400054

Tween 20	Bio-Rad	Cat# 1706531
BSA	Sigma Aldrich	Cat# A7906
RNeasy kit	QIAGEN	Cat# 74104
iScript cDNA synthesis kit	Bio-Rad	Cat# 1708891
iTaq Universal SYBR Green PCR Kit	Bio-Rad	Cat# 1725121
Formamide	Sigma Aldrich	Cat# F9037-100ML
TURBO DNase	Invitrogen	Cat# AM2238
10% Formalin fixative	VWR	Cat# 16004-121
HBSS	Thermo Fisher Scientific	Cat# 14060040
RNase A	Invitrogen	Cat# AM2271
Proteinase K	Invitrogen	Cat# 25530049
PBS	Sigma Aldrich	Cat# P5493
RIPA buffer	Thermo Fisher Scientific	Cat# 89900
MTT	Sigma Aldrich	Cat# M5655
